# The bile acid receptor FXR attenuates acinar cell autophagy in chronic pancreatitis

**DOI:** 10.1038/cddiscovery.2017.27

**Published:** 2017-06-19

**Authors:** Xiaodong Zhou, Li Xie, Frank Bergmann, Volker Endris, Oliver Strobel, Markus W Büchler, Guido Kroemer, Thilo Hackert, Franco Fortunato

**Affiliations:** 1 Department of General, Visceral and Transplantation Surgery, University Clinic Heidelberg, Heidelberg, Germany; 2 Section Surgical Research, University Clinic Heidelberg, Heidelberg, Germany; 3 Affiliated People's Hospital of Jiangsu University Zhenjiang, Jiangsu, China; 4 Institute of Pathology, University Clinic Heidelberg, Heidelberg, Germany; 5 Equipe 11 labellisée par la Ligue contre le Cancer, Centre de Recherche des Cordeliers, Paris, France; 6 INSERM, U1138, Paris, France; 7 Université Paris Descartes, Sorbonne Paris Cité, Paris, France; 8 Université Pierre et Marie Curie, Paris, France; 9 Pôle de Biologie, Hôpital Européen Georges Pompidou, AP-HP, Paris, France; 10 Cell Biology and Metabolomics platforms, Gustave Roussy Cancer Campus; Villejuif, France; 11 Karolinska Institute, Department of Women's and Children's Health, Karolinska University Hospital, Stockholm, Sweden

## Abstract

The functional relationship between bile acid (BA) and autophagy has not been evaluated in the context of pancreatitis. Here we investigated whether BA and their nuclear farnesoid X receptor (FXR) modulate autophagy and the development of pancreatitis. FXR expression, autophagy, apoptosis and necroptosis were determined in human chronic pancreatitis (CP) tissue *in vivo* and in pancreatic cells lines *in vitro* by means of real-time PCR, immunoblots and immunofluorescence. Pancreatic cell lines exposed to the most abundant BAs glycochenodeoxycholate (GCDC) and taurocholic acid (TCA) increased the expression of nuclear FXR and diminished that of the essential autophagy-related protein ATG7. BA was also elevated in pancreatic tissues from CP patients, correlating with elevated FXR and curtailed ATG7 expression with locally reduced autophagic activity. This was accompanied by an increased manifestation of CP hallmarks including apoptosis, necroptosis, inflammation and fibrosis. The present results suggest a cascade of events in which local accumulation of BA signals via FXR to suppress autophagy in pancreatic acinar cells, thereby unleashing acinar cell apoptosis and necroptosis. Thus, BA may cause CP by suppressing autophagy and exacerbating acinar cell apoptosis and necroptosis.

## Introduction

The nuclear bile acid receptor, known as farnesoid X receptor (FXR), plays an essential role in regulating the metabolism of BAs, lipids and glucose.^1,2^ Similarly, nutritional and energy homeostasis is largely influenced by the evolutionarily conserved autophagy pathway.^3,4^ Activation of FXR can suppress the activation of autophagy by diminishing the expression of several autophagy-related genes such as Atg7 and Lamp-2,^5,6,7^ suggesting a link between BA, their action on FXR, autophagy and metabolic regulation. Autophagy is activated under cellular stress or upon energy depletion in order to sequester cytoplasmic components for their degradation/recycling and energy generation.^8,9^ Pancreatic acinar cells are highly efficient in synthesizing and releasing digestive enzymes, meaning that they are continuously exposed to high levels of misfolded or denatured proteins with potentially toxic functions. For this reason, pancreatic acinar cells must quench latent cellular damage by means of autophagy. Disabled autophagy has been linked to multiple distinct pathologies including inflammatory diseases such as pancreatitis.^9,10,11^ Thus, depletion of pancreatic Atg7 or Atg5 (which both are essential for the autophagic process) can induce acinar cell death, thereby triggering acute pancreatitis (AP) and later chronic pancreatitis (CP).^10,12,13^

Autophagy has previously been considered to be mostly regulated by post-transcriptional modification of pre-existing proteins to maintain energy status in rapidly changing conditions. However, transcriptional regulators such as NFкB, FOXO3 and STAT3 can repress autophagy activity under different metabolic conditions.^7, 14,15,16,17, 18,19^ Moreover, recent data indicate that autophagy also participates in the transcriptional regulation of a large numbers of genes that determine cellular energy status.^3^

Gallstones or bile acid (BA) reflux into the pancreas duct are common etiologies for biliary pancreatitis. BA has also been shown to induce acinar cell death by reducing mitochondrial membrane potential, increasing reactive oxygen species and depletion of energy, all of which are known to promote acinar cell apoptosis and necrosis in pancreatitis.^20,21,22,23,24^ In humans, the most abundant primary BA are glycochenodeoxycholic acid (GCDC) and taurocholic acid (TCA). Both GCDC and TCA can induce necrosis in primary human hepatocytes, reflecting focal liver necrosis occurring in patients with obstructive cholestasis.^25^ Similarly, retrograde glycodeoxycholic acid (GDOC) infusion into the pancreatic duct induces experimental necrotizing pancreatitis in rodents.^26,27,28,^ This appears clinically relevant because gallstone obstruction of the Ampulla of Vater (which forms by the junction of the common bile duct and the pancreatic duct) causes Pancreatitis in humans.^29,30^

In view of the fact that FXR acts as a major repressor for autophagy signaling,^1,2^ it is tempting to speculate that FXR might subvert the autophagy activity that usually counteracts development and progression of pancreatitis. Beyond FXR, BA act on the G-protein-coupled cell surface bile acid receptor (Gpbar1). Gpbar1-deficient mice exhibit diminished AP severity following retrograde infusion of taurolithocholic acid (TLCS).^31^ However, in mice, Fxr-deficiency did not affect the severity of AP induced by caerulein, and patients with AP do not show genetic variations in the FXR locus. Hence, the role of FXR in the development of human CP remains elusive.^32^

Driven by the aforementioned premises, we decided to investigate the possible link between BAs, activation of the BA receptor FXR and downregulation of autophagy. Here, we report that human CP specimens exhibit an increase in BA and nuclear FXR expression, loss of ATG7 protein, and consequent reduced autophagy activity in acinar cells. Our clinical data (on human CP specimens) and experimental *in vitro* results on pancreatic cell lines are in line with the idea that autophagy inhibition facilitates acinar cell death by necroptosis and apoptosis, thereby triggering inflammation and fibrosis.

## Results

### Bile acid increased nuclear FXR and suppressed ATG7 expression *in vitro*

**Figure 1:**
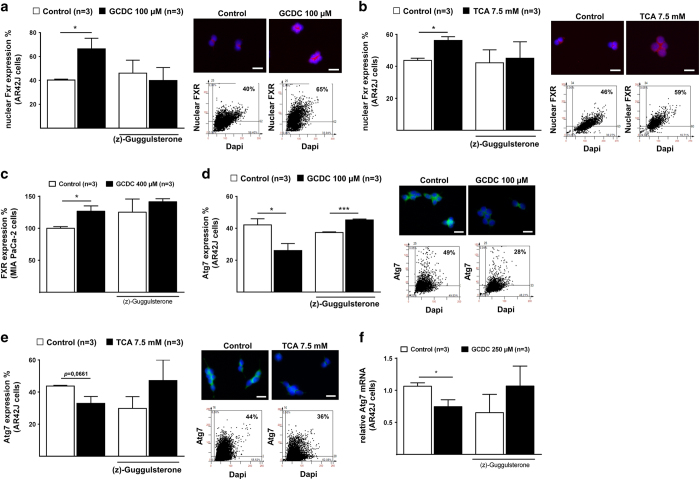
Bile acid activates Fxr and decreased Atg7 expression in pancreatic cell lines. (**a**) Representative IF FACS-like quantitation and scattergrams of nuclear FXR in rat AR42J cells exposed to 100 *μ*M GCDC with or without (z)-Guggulsterone (GS) stained for DAPI (blue) and FXR (red). Nuclear FXR expression values obtained from Table 1 were plotted as means±S.E.M. for three independent experiments as indicated in the graphs. (**b**) Similar to A, cells were exposed to 7.5 mM TCA with or without (z)-Guggulsterone. (**c**) Representative IF FACS-like quantitation of FXR in human MIA PaCa-2 cells to 400 *μ*M GCDC with or without (z)-Guggulsterone (GS). (**d**) Representative IF FACS-like quantitation and scattergrams of Atg7 in rat AR42J cells exposed to 100 *μ*M GCDC with or without (z)-Guggulsterone stained for DAPI (blue) and Atg7 (green). Atg7 expression values obtained from Table 1 were plotted as means±S.E.M. for three independent experiments as indicated in the graphs. (**e**) Similar to D, cells were exposed to 7.5 mM TCA with or without (z)-Guggulsterone. (**f**) Real-time PCR confirmed that rat AR42J cells exposed to GCDC attenuated mRNA Atg7 level and the Fxr antagonist GS prevented Atg7 mRNA suppression. Normalized ∆∆CT values of Atg7 mRNA expression were plotted as means±S.E.M. for the numbers of experiments indicated in the graphs. *P<0.05; ***P<0.001; 20×objective; scale bar=20 *μ*m.

The bile acid (BA) components GCDC and TCA are cytotoxic to hepatocytes and rat pancreatic acinar-like AR42J cells.^2,25^ Culture in the presence of either GCDC or TCA increases the expression level of nuclear FXR in AR42J cells. This effect was suppressed by the FXR antagonist (z)-Guggulsterone (GS) (Figure 1a and b, Table 1). Similar, human pancreatic MIA PaCa-2 cancer cell lines increased FXR in response to GCDC (Figure 1c). Surprisingly, GCDC and TCA exposure significantly decreased the expression of Atg7 protein in an Fxr-dependent fashion, meaning that GS abolished the negative effects of GCDC and TCA on Atg7 expression in AR42J cells (Figure 1d and e, Table 1). Quantitative real-time PCR confirmed that GCDC incubation attenuate the mRNA expression ATG7 (Figure 1f). In addition Human BxPc-3 cells also attenuate ATG7 expression after GCDC treatment (Supplementary Figure A). Quantitative real-time PCR of human MIA PaCa-2 (Supplementary Figure B) and rat acinar-like AR42J cells (Supplementary Figure C) revealed an increase of Fxr mRNA and a decrease of Atg7 and Atg5 mRNA in response to GCDC, confirming our results. Altogether, these data indicate that BA facilitate the expression and/or nuclear translocation of FXR and suppress the transcription of ATG7 in pancreatic acinar cells, confirming similar findings in hepatocytes.^3,6^

### Bile acid increased nuclear FXR and suppressed ATG7 in acinar cells from human chronic pancreatitis tissue

**Figure 2:**
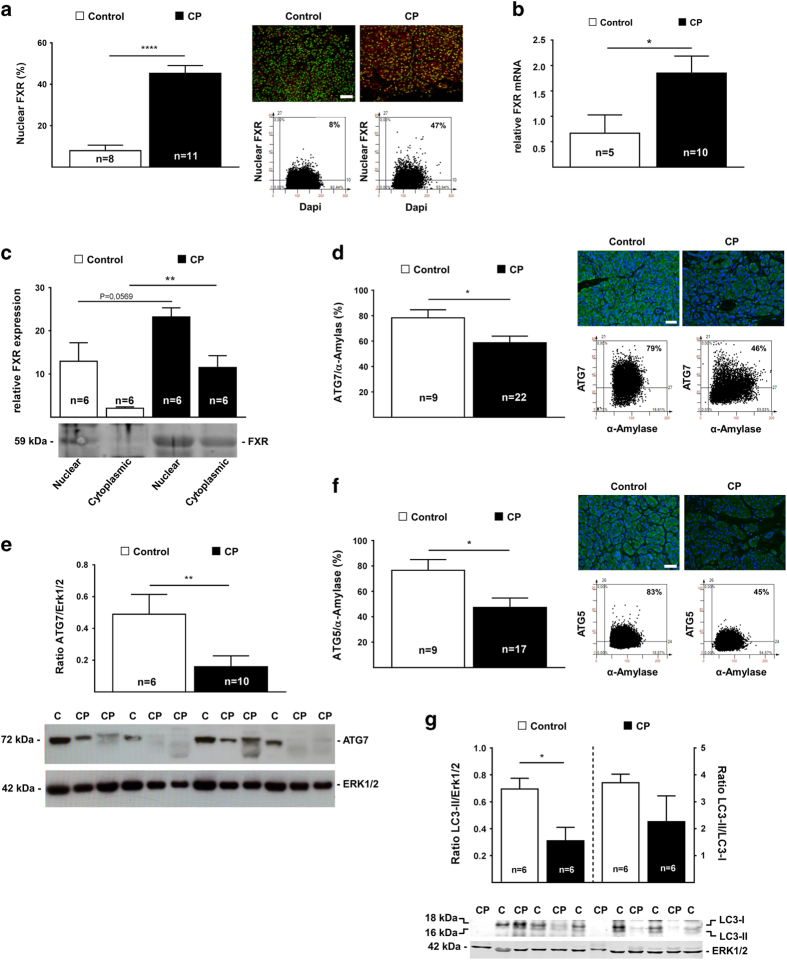
Human chronic pancreatitis tissue exhibited increased of FXR and a loss of ATG7 with reduced autophagy activity. (**a**) Representative IF FACS-like quantitation and scattergram of nuclear FXR in human pancreatic tissue stained for DAPI (green) and FXR (red). Nuclear FXR expression values obtained from Table 2 were plotted as means±S.E.M. for the numbers of patients indicated in the graphs. (**b**) Real-time PCR confirmed that FXR mRNA is significantly increased in human pancreatic tissue. Normalized ∆∆CT values of ATG7 mRNA expression were plotted as means±S.E.M. for the numbers of experiments indicated in the graphs. (**c**) Representative immunoblot autoradiograph and analysis. Human pancreatic tissue was homogenized and fractionated into nuclear and cytoplasmic fractions and were subject for FXR immunoblot assay. Relative fragment intensity were plotted as means±S.E.M. (**d**) Representative IF FACS-like quantitation and scattergrams of ATG7 in human pancreatic tissue stained for DAPI (blue) ATG7 (green) and *α*-Amylase (for visibility not shown). ATG7 expression values obtained from Table 2 were plotted as means±S.E.M. for the numbers of patients indicated in the graphs. (**e**) Representative immunoblot autoradiograph and analysis of the ratio of ATG7 and ERK1/2 in human pancreatic tissue. Ratios were plotted as means±S.E.M. for the numbers of animals indicated in the graphs. (**f**) Representative IF FACS-like quantitation and scattergrams of ATG5 in human pancreatic tissue stained for DAPI (blue) ATG5 (green) and *α*-Amylase (for visibility not shown). ATG5 expression values obtained from Table 2 were plotted as means±S.E.M. for the numbers of patients indicated in the graphs. (**g**) Representative immunoblot analysis of reduced conversion of LC3-I to LC3-II in human CP tissue. Loss of ATG7 inhibited the conversion of LC3-1 to LC3-II, determined by WB analysis using the ratio of LC3-II and LC3-I and the ratio of LC3-II and Erk1/2. Ratios were plotted as means±S.E.M. for the numbers of patients indicated in the graphs. *P<0.05; **P<0.01; ****P<0.0001; 20×objective; scale bar=20 *μ*m.

We next determined whether BA might be involved in the pathogenesis of human CP. Quantitative immunofluorescence (IF) on human tissue sections from healthy controls and CP patients revealed a 5.7-fold increase in the expression of bile acid receptor FXR in acinar cell nuclei (Figure 2a, Table 2). Quantitative real-time PCR also confirmed increased FXR mRNA expression in chronic pancreatitis tissue (Figure 2b). Tissue fractionation into nuclear and cytoplasmic fraction confirmed an augmentation in nuclear FXR in CP samples, confirming the results obtained by IF and real-time PCR (Figure 2c).

Similar to our *in vitro* results, ATG7 expression was significantly reduced in acinar cells from CP patients compared to controls, as determined by quantitative IF (Figure 2d, Table 2). Moreover, immunoblot of tissue homogenate confirmed a significantly attenuated expression of ATG7 in CP patients compared to heathy controls (Figure 2e). ATG5 was also significantly reduced in acini from CP patients compared to heathy controls, as determined by quantitative IF (Figure 2f, Table 2).

We next investigated additional autophagy marker in CP tissue. As compared to healthy control tissues, human CP specimens were characterized by a reduced abundance of the lipidated from of LC3 (LC3-II) (Figure 2g) and an accumulation of the autophagic substrate sequestosome-1 (STQM1/p62) (Supplementary Figure D, Table 2), indicating an overall reduced autophagy activity in human chronic pancreatitis samples. Quantitative real-time PCR confirmed reduced mRNA levels of ATG7, ATG5, LC3 and Beclin-1 in CP patients, providing additional evidence of reduced autophagy signaling in CP specimens (Supplementary Figure E). FOXO3 represses the expression of several autophagy genes including ATG7 and BECLIN-1.^3,6^ Cytoplasmic FOXO3 levels were upregulated in CP tissues (Supplementary Figure F), in line with the possibility that BA-induced both FXR and FOXO3, which in turn attenuate autophagy activity in CP. In contrast, STAT3, which is another transcription factor that can inhibit autophagy signaling,^7^ did not show any alteration in CP (data not shown). Quantitation values of FXR and autophagy are summarized in Table 2. Altogether, these results suggest reduced acinar cell autophagy activity in human CP.

### Human chronic pancreatitis is associated with increased apoptosis and necroptosis

**Figure 3:**
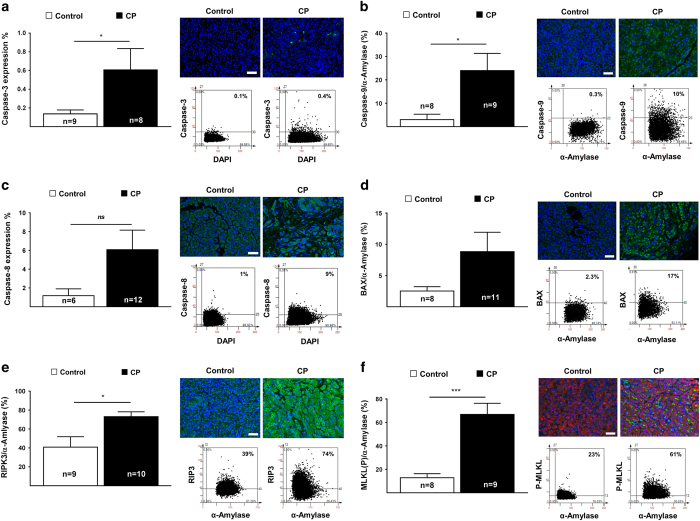
Loss of ATG7 is associated with activation of apoptosis and necroptosis in human chronic pancreatitis tissue. (**a**) Reduced autophagy facilitates increased pancreatic caspase-3 in human CP tissue, determined by FACS-like IF quantitation, stained for DAPI (blue) and active caspase-3 (green) for the numbers of patients indicated in the graphs. Caspase-3 expression values were plotted as means±S.E.M. for the numbers of patients indicated in the graphs. (**b**) Increase of caspase-9 expression in human CP tissue, determined by FACS-like IF quantitation and scattergrams, stained for DAPI (blue) and active caspase-9 (green). Caspase-9 expression values obtained from Table 3 were plotted as means±S.E.M. for the numbers of patients indicated in the graphs. (**c**) Increase of caspase-8 expression in human CP tissue, determined by FACS-like IF quantitation and scattergrams, stained for DAPI (blue) and caspase-8 (green). Caspase-8 expression values were plotted as means±S.E.M. for the numbers of patients indicated in the graphs. (**d**) Increase of pro-apoptotic Bax expression in human CP tissue, determined by FACS-like IF quantitation and scattergrams, stained for DAPI (blue) and Bax (green). Bax expression values obtained from Table 3 were plotted as means±S.E.M. for the numbers of patients indicated in the graphs. (**e**) Increased RIP3 expression in human CP tissue, determined by FACS-like IF quantitation and scattergrams, stained for DAPI (blue), RIP3 (green) and *α*-Amylase (for visibility not shown). Representative and RIP3 expression values obtained from Table 3 were plotted as means±S.E.M. for the numbers of patients indicated in the graphs. (**f**) Increased phosphorylated MLKL expression in human CP tissue, determined by FACS-like IF quantitation and scattergrams, stained for DAPI (blue), MLKL(P) (green) and *α*-Amylase (red). MLKL(P) expression values obtained from Table 3 were plotted as means±S.E.M. for the numbers of patients indicated in the graphs. *P<0.05; ***P<0.001; 20× objective; scale bar=20 *μ*m.

We next investigated markers of apoptosis and necroptosis in human CP samples by means of quantitative IF. Cleaved caspase-3 (Figure 3a) and caspase-9 (Figure 3b) increased significantly in acinar cells from CP tissues compared to controls. Caspase-8 and Bax, were also elevated in CP tissues (Figure 3c and d), indicating activation of apoptosis in pancreatic acinar cells. We next determine the activity of necroptosis in pancreatitis. The expression of RIP3 (Figure 3e) and phosphorylated MLKL (Figure 3f) were also increased in acinar cells from CP patients. Hyperphosphorylation of MLKL was confirmed by immunoblot (Supplementary Figure G), supporting the idea that necroptosis is activated in human CP as well. Quantitation values of apoptosis and necroptosis are summarized in Table 3.

### Increased tissue bile acid is associated with pancreatic pathology and chronic pancreatitis

**Figure 4:**
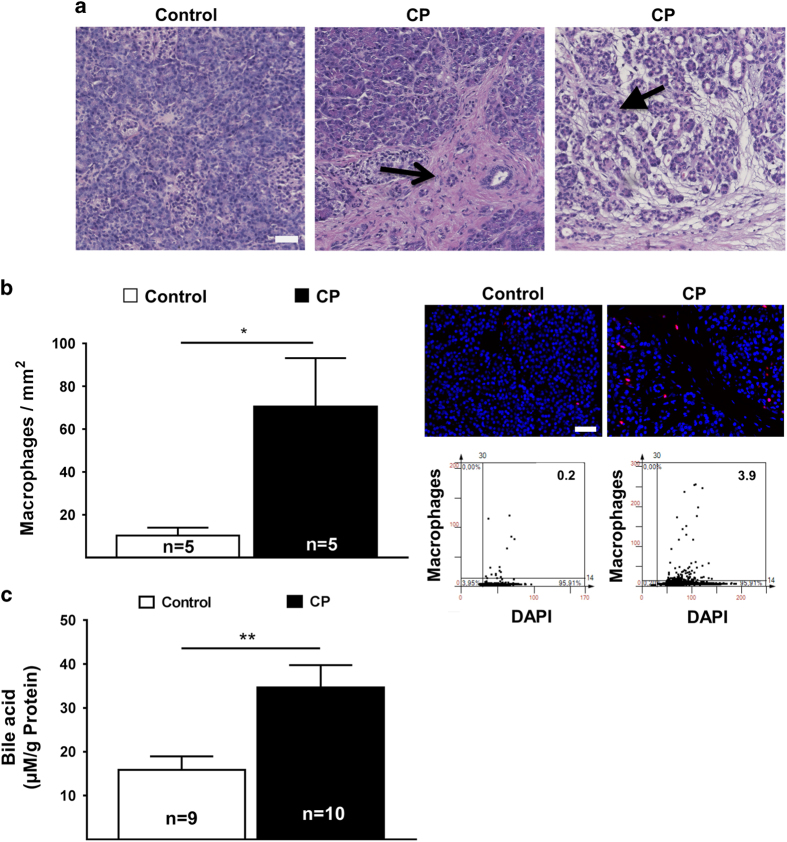
Chronic pancreatitis with elevated local bile acid and with FOXO3 expression. (**a**) Representative H&E staining of human chronic pancreatic tissue and histopathology score. Open arrow indicate fibrosis, closed arrow indicate destructed tissue with signs of ADMs (20× objective; Scale bar, 20 *μ*m). (**b**) Increased of macrophage infiltration in human CP tissue, determined by FACS-like IF quantitation and scattergram, stained for DAPI (blue), macrophage marker (red) in control and chronic pancreatitis patients. Macrophage numbers were plotted as means±S.E.M. for the numbers of patients indicated in the graphs. (**c**) Bile acid concentration was plotted as means±S.E.M. for the numbers of human tissue extract as indicated in the graphs. *P<0.05; **P<0.01; 20× objective; scale bar=20 *μ*m.

We next evaluated pancreatic specimens from CP patients for inflammation and fibrosis. Hematoxylin-eosin staining severity assessment revealed an expected increase in fibrosis and in inflammation (Figure 4a). Occasionally acinar-to-ductal cell metaplasia (ADM) and pancreatic intraepithelial neoplasia (PanIN), which may develop from ADM, can be observed (Table 4).^33^ Macrophage infiltration was highly elevated in the pancreata from CP patients compared to healthy controls (Figure 4b), perhaps as an attempt to clear apoptotic and necroptotic acinar cells. We next determined bile acid content in pancreatic tissue using newly developed bile acid assay. Bile acid concentrations increased significantly in CP tissues compared to healthy pancreata (Figure 4c), suggesting that BA can facilitate acinar cell cytotoxicity, tissue injury and pancreatitis.

## Discussion

In the present report, we provide evidence that BA participates in the development of pancreatitis by activating acinar cell FXR, leading to suppressed levels of acinar cell autophagy, thereby triggering acinar cell death, inflammation and fibrosis resulting in CP. BA has a strong etiological role in human pancreatitis,^3,6^ and retrograde BA infusion is sufficient to trigger pancreatitis in animal models.^30,31,34^ BA has recently been reported to stimulate ATP release by exocrine acinar cells, thus compromising the cellular energy status.^2^

The bile acid receptor FXR has been described as one of the effective transcription factors that suppresses ATG7, thus disabling the autophagic machinery in hepatocytes.^25^ Indeed, BA concentrations are increased and FXR is highly activated in acini from human CP specimens, as well as in human and rat pancreatic cell lines exposed to GCDC *in vitro*, in line with the possibility that BA and FXR activation contributes to the development of human CP. FOXO3 has been reported to repress the expression of genes required for autophagy as well,^7^ and pancreatic specimens from patients with CP indeed contained elevated levels of FOXO3, suggesting that both FXR and FOXO3 contribute to the inhibition of autophagy in CP.

Loss of pancreatic Atg7 has been reported to be sufficient to induce massive acinar cell death associated with pancreatitis. Conditional knockout of Atg7 in pancreatic cells caused local inactivation of the autophagy machinery with a consequent loss of acinar cells mass, pancreatic atrophy, inflammation and fibrosis.^9,10, 35,36,37^ Importantly, human pancreatic ATG7 mRNA and protein levels were significantly reduced in clinical specimens from CP patients, suggesting that the pathogenesis of human CP may be linked to the reduction of pancreatic ATG7 and overall reduced pancreatic autophagy. Accordingly, loss of acinar cell ATG7 promoted acinar cell apoptosis and necroptosis in human CP specimens. Necroptosis or regulated necrosis has potential clinical relevance since *Rip3*^−/−^ mice are protected from experimental caerulein-induced pancreatitis.^38^ As a result, it appears plausible that the enhanced expression of RIP3 coupled to the hyperphosphorylation of its substrate MLKL that we observed in human CP specimens reflects a pathophysiologically relevant event of necroptotic signaling.

The quantitative IF technique used in the present study allows the measurement of tissue protein expression levels in distinct areas of the tissue, comparing the expression level of proteins in apparently intact zones, adjacent to inflammatory lesions, as well as in exhausted pancreatic tissue areas. Such exhausted areas were not observed in the CP specimens included in this study. Thus, the signs of disabled autophagy (reduced ATG7 and accumulated STQM1/p62) and the concomitant upregulation of apoptotic and necroptotic markers are unlikely to result from massive tissue destruction or exhaustion.

Altogether, our results suggest a molecular cascade of events in which BA causes the upregulation and nuclear translocation of their receptor FXR, thereby stimulating the transrepression of the essential autophagy-relevant gene *ATG7* in pancreatic acinar cells. Local inactivation of the autophagic machinery then compromises the survival of pancreatic exocrine acinar cells that activate a diverse array of cell death mechanisms including apoptotic and necroptotic pathways. This process is followed by invasion of the pancreas by inflammatory leukocytes from the myeloid lineage, fibrogenesis resulting in insufficiency of the exocrine and/or endocrine function of the pancreas. Given that patients with AP and CP are affected by high mortality rates and that no appropriate treatments are available for their therapeutic management, future clinical studies should explore the stimulation of autophagy as a possible strategy of reducing the mortality of AP and CP.

## Materials and methods

### Antibodies and reagents

Antibodies were selected according to proven functionality for formalin-fixed paraffin-embedded (FFPE) tissue sections and WB by the seller or by publication records. The following antibodies were used for WB: Erk2 (sc-154), hu ATG7 (sc-8668), p62 (sc-25575) and Bax (sc-526), all were purchased from Santa Cruz Biotechnology (Heidelberg, Germany). Human Rip3 (ab72106) and MLKL (ab194699) were purchased from Abcam (Cambridge, UK). LC3 (5F10, 0231-100) was purchased from Nanotools (Teningen, Germany). Atg5 (AP1812a) was purchased from ABGENT (San Diego, USA).

For IF we used the following antibodies: FXR (sc-13063), FOXO3 (sc-34897), p62 (sc-25575), Bax (sc-526) and hu *α*-Amylase (sc-46657) all were purchased from Santa Cruz Biotechnology (Heidelberg, Germany). MPO (ab9535), hu Rip3 (ab152130), hu MLKL (phospho S358)(ab187091), HMGB1 (ab18256) and Macrophage Marker (sc-66204) were purchased from Abcam (Cambridge, UK). F4/80 (NBP2-12506), Active/cleaved Caspase-8 (NB100-56116), active/cleaved Caspase-9 (NB100-56118) and hu ATG7 (NBP1-40039) was obtained from Novus Biologicals (Cambridge, UK). Cleaved Caspase-3 (cs-9661) was purchased from Cell Signaling Technology (Danvers, USA). ATG5 antibody used in IF was same as WB listed above. Secondary anti-rabbit Cy3-, or Cy5-conjugated and anti-mouse Cy3-, or Cy5-conjugated antibodies were purchased from Medac GmbH (Wedel, Germany) and applied for IF.

Secondary goat anti-rabbit IgG-HRP (sc-2054), goat anti-mouse IgG-HRP (sc-2055) and donkey anti-goat IgG-HRP (sc-2020) were purchased from Santa Cruz Biotechnology (Heidelberg, Germany). In addition IRDye 680RD Goat anti-mouse (926-68070) and IRDye 800CW goat anti-rabbit (926-32211) obtained from LI-COR (Bad Homburg, Germany) were used for IB. All other chemicals were from Sigma-Aldrich (Deisenhofen, Germany), if not stated otherwise.

### Human subjects

The study was approved and renewed in August 2012 by the Ethics Committee of the University Medical Faculty of Heidelberg. All patients signed the consent form and were informed that their tissue will be used in research. Tissue samples from 17 chronic pancreatitis patients (8 females, 9 males, median age 45 years) and 10 healthy donor control tissue (5 females, 5 males, median age 54 years) (5 CP and 1 control were not histopathologic evaluated) were randomly collected from patients visiting the Department of Surgery of our University. Normal pancreatic tissue samples were provided from healthy organ donors when there was no appropriate recipient for transplantation. Surgical freshly removed tissue samples were immediately either fixed in 4% buffered formalin or snap frozen in liquid nitrogen and stored at −80 °C for protein extraction.

### Pancreatic histopathology

FFPE pancreatic tissue were cut into 4 *μ*m thick sections and stained with hematoxylin & eosin (H&E). Histopathological evaluation of the pancreatic tissue included: the severity of fibrosis, the severity of inflammation, the activity of inflammation, and the severity and activity of the perineural inflammation, as described recently.^39^ The severity of fibrosis was determined by the modified evaluation described by Ammann and colleagues,^40^ using a scoring system based on focal *versus* diffuse extension of intralobular and perilobular fibrosis. The severity and distribution of fibrosis in the investigated specimens were graded according to a scoring system shown in Table 4. Severity of fibrosis was determined by the use of an intralobular and perilobular fibrosis score of: mild 0 (0–4), moderate I (5–9) or severe II (10–12) fibrosis.

The severity of inflammation was scored as absent (0), mild (I), moderate (II) or severe (III), based on the determination of the overall accumulation of inflammatory cells (lymphocytes, plasma cells and macrophages). The activity of inflammation was based on the presence and density of neutrophil granulocytes representing the early inflammatory response. These were scored as absent (0), mild (I) or moderate to severe (II). Overall severity of inflammation was scored as absent (0), mild (0–1), moderate (2–3) or severe (4–6). Acinar-to-ductal metaplasia (ADM) was scored according to the tissue frequency, with scores of absent (0), mild (I) or moderate to severe (II). Mice pancreatic tissue was scored very similarly.

### Immunofluorescence

IF was conducted using 4 *μ*m thin FFPE pancreatic tissue sections obtained from Atg7 mice and human specimens and processed, as described in detail previously.^9,41,42^ All images were processed using the StrataQuest software (TissueGnostics), allowing the quantitation of the total cell numbers from DAPI-positive cells, as well as quantitation of target positive cells. In FACS-like scattergrams, the cells were plotted according to their Cy3 and Cy5 IF intensity *versus* their DAPI-intensity from the entire tissue. IF-positive cells were gated in the scattergrams according to negative controls (no primary antibody), and the fluorescence intensity was expressed as a percentage of the mean intensity of the DAPI staining and mean intensity of the target protein staining in a FACS-like scattergram approach, as described previously.^9,41,42^ Infiltrated macrophages or MPO-positive monocytes were determined by the numbers of positive cell per mm^2^ tissue.

### Immunoblot analysis

Immunoblot analysis was performed in order to evaluate variations in the expression of specific proteins involved in apoptosis, autophagy and necrosis signaling. Human frozen tissues were homogenized on ice, as described previously.^9^ Protein loading control was performed with Erk-2 (after using a Restore Western Blot Stripping Buffer Plus (Pierce Biotech., Rockford, US) to ensure equal protein loading, according to the instructions. Protein fragments were processed and analyzed by using computer-assisted software ImageJ.

### Pancreatic tissue organelle fractionation

Pancreatic tissue (*n*=12) was fractionated according to our previously described method.^9^ Protein concentrations in nuclear and cytoplasmic fractions were determined by the BCA protein assay procedure (Pierce, Rockford, IL, USA) and both fractions were processed and analyzed as described for whole tissue homogenate.

### Real-time PCR

Frozen whole human pancreatic tissues were extracted using TRIzol-Reagent (Life Technologies, Invitrogen) and processed, as described previously.^41,42^ The primer sequences for human and rat have been previously reported and were additionally checked using NCBI primer blast.^43–45^

### Primer sequences in real-time PCR analysis


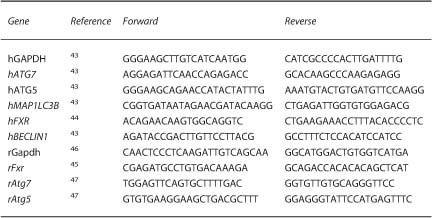


### *In vitro* cell culture

Human MIA PaCa-2 and BxPC-3 pancreatic cancer cell lines and rat AR42J pancreatic acinar-like cancer cell line were obtained from American Type Culture Collection (Manassas, USA) and were grown in DMEM, RPMI 1640 or Hams´ F-12 medium supplemented with either 10 or 20% heat-inactivated FCS, 50 U/ml penicillin G, 50 *μ*g/ml streptomycin and 2.5 *μ*g/ml Plasmocin. They were maintained at 37 °C in a humidified atmosphere of 95% air and 5% CO_2_ until plating for the bile acid studies. Ten-thousand cells were grown on Ibidi *μ*-Silde 8 well chamber slides (Ibidi, Germany) for 24 h followed by the treatment with GCDC or TCA with or without GS. Twenty-four hours after the treatment, the cells were fixed in ice cold aceton for 10 min at RT, followed by immunofluorescence staining, as described for the tissue. Briefly, fixed cells were washed with PBS and blocked with 5% goat serum, for 1 h at RT. Primary human or rat anti-FXR, anti-Atg7 and anti-Atg5 antibodies were incubated overnight at 4 °C in a dark humidified chamber, and stained with either anti-rabbit-Cy5 and/or anti-mouse-Cy3 labeled secondary antibodies for 1 h at RT, followed by several steps of washing and incubation with DAPI for 20 min. The wells were mounted in ProLong Gold Antifade Reagent (Molecular Probes). Images were captured and analyzed using the TissueFaxs microscope unit (TissueGnostics), as described for the immunofluorescence approached.

### Bile acid assay

Total bile acid was determined using the total BA assay kit according to the instructions (Cell Biolabs Inc., San Diego, USA).

### Statistical analysis

Statistical analysis was performed using ANOVA, followed by Student’s *t-Test* for each group. For all column data sets, analysis of identified outliers was performed and results were considered significant when *P* value ⩽0.05, indicated with *, using GraphPad Prism 6 software for statistical calculations. The *in vitro* results were normalized. All results were reported as mean±S.E.M. (standard error of the mean) as indicated with the significance score (*<0.05; **<0.01; ***<0.001, ****<0.0001) in the figure legend.

## Figures and Tables

**Table 1:** Bile acid exposure increased Fxr and decreased Atg7 expression

*Fxr*	*Control n=3*		*GCDC=3*		
		%		%	*P* value
Total cells	15 348	100	14 345	100	
Fxr	6247	40.7	9208	64.2	0.0433
					
*Fxr*	*GS n=3*		*GCDC+GS=3*		
		%		%	*P* value
Total cells	8937	100	11 018	100	
Fxr	3488	39	3498	31.7	ns
					
*Fxr*	*Control n=3*		*TCA=3*		
		%		%	*P* value
Total cells	12 658	100	8361	100	
Fxr	5487	43.3	4496	53.8	0.0107
					
*Fxr*	*GS n=3*		*TCA+GS=3*		
		%		%	*P* value
Total cells	9740	100	5279	100	
Fxr	5033	51.6	2614	49.5	ns
					
*Atg7*	*Control n=3*		*GCDC=3*		
		%		%	*P* value
Total cells	10 245	100	9137	100	
Fxr	4163	40.6	2465	27	0.0497
					
*Atg7*	*GS n=3*		*GCDC+GS=3*		
		%		%	*P* value
Total cells	8450	100	6565	100	
Fxr	3180	37.6	2966	45.1	0.0004
					
*Atg7*	*Control n=3*		*TCA=3*		
		%		%	*P* value
Total cells	17 467	100	11 984	100	
Fxr	7676	43.9	4102	34.2	0.0661
					
*Atg7*	*GS n=3*		*TCA+GS=3*		
		%		%	*P* value
Total cells	15 611	100	11 136	100	
Fxr	4359	27.9	5350	48	ns

Table 1 summarizes the number of positive stained cells for Fxr or Atg7 in response to GCDC or TCA with or without GS for three independent experiments. Cell numbers were identified and count using StataQuest analysis software (TissueGnostics), and expressed as % as described in the methods.

**Table 2:** Increased FXR suppress autophagy in human chronic pancreatitis

*FXR*	*Control: n=8*		*CP: n=11*		
		%		%	*P value*
Total cells	4 582 670	100.00	3 197 789	100.0	
α-Amylase	4 139 898	90.34	1 937 714	60.6	0.0005
FXR	288749	6.30	967 605	30.3	0.0491
FXR/α-Amylase	278 241	6.72	841 495	43.4	<0.0001
					
*ATG7*	*Control: n=9*		*CP: n=22*		
		%		%	*P value*
Total cells	3 540 686	100	3 698 992	100	*–*
α-Amylase	3 286 157	93	2 605 567	70	0.0198
ATG7	2 732 982	77	1 759 413	48	0.0023
ATG7/α-Amylase	2 636 007	80	1 425 548	55	0.0414
					
*ATG5*	*Control: n=9*		*CP: n=17*		
		%		%	*P value*
Total cells	3 520 469	100	2 491 868	100	*–*
α-Amylase	3 250 823	92	1 951 276	78	0.0239
ATG5	2 388 958	67	859 164	24	0.0013
ATG5/α-Amylase	2 304 604	70	762 979	39	0.0212
					
*p62*	*Control: n=5*		*CP: n=15*		
		%		%	*P value*
Total cells	2 821 348	100	2 698 781	100	*–*
α-Amylase	2 410 310	86.1	2 016 326	65.2	ns
p62	270 934	11.7	603 160	19.4	0.0240
p62/α-Amylase	265 793	1.4	561 495	30.3	0.0107

Table 2 summarizes the number of Dapi positive stained human pancreatic acinar cells in the tissue form controls or chronic pancreatitis tissue. Cell numbers were identified by Dapi staining and the expression level for FXR, ATG7, ATG5 and p62 were determined using StataQuest analysis software (Tissue Gnostics), and expressed as % from total or *α*-amylase cells as described in the methods.

**Table 3:** Decreased autophagy promotes apoptosis and necroptosis in human chronic pancreatitis

*CASPASE-9*	*Control: n=7*		*CP: n=9*		
		%		%	*P value*
Total cells	1 606 731	100	1 067 090	100	
α-Amylase	1 467 096	91.3	899 865	84.3	ns
Caspase-9	45 620	2.8	167 716	15.7	0.0244
Casp-9/α-Amylase	45 341	3.1	157 752	17.5	0.0216
					
*BAX*	*Control: n=8*		*CP: n=11*		
		%		%	*P value*
Total cells	934 430	100	2 154 365	100	
α-Amylase	791 991	84.8	1 279 723	59.4	0.0131
BAX	18 168	1.9	101 674	4.7	ns
BAX/α-Amylase	18 012	2.3	87 573	6.8	ns
					
*RIP3*	*Control: n=9*		*CP: n=10*		
		%		%	*P value*
Total cells	1 665 829	100	990 111	100	–
α-Amylase	1 525 552	92	614 947	62	0.0077
RIPK3	626 437	38	592 351	60	ns
RIPK3/α-Amylase	608 111	40	452 491	74	0.0138
					
*MLKL*	*Control: n=8*		*CP: n=9*		
		%		%	*P value*
Total cells	2 176 951	100	1 684 776	100	–
α-Amylase	1 905 447	87	1 169 053	59	ns
MLKL	283 385	13	949 673	43	0.0214
MLKL/α-Amylase	250 265	13	882 427	66	0.0001

Table 3 summarizes the number of Dapi positive stained human pancreatic acinar cells within the tissue form controls or chronic pancreatitis tissue. Cell numbers were identified by Dapi staining and the expression level for Caspase-9, BAX, RIP3 and pMLKL were determined using StataQuest analysis software (TissueGnostics), and expressed as % from total or *α*-amylase cells as described in the methods.

**Table 4:** Histopathology score of human chronic pancreatitis tissue

*Control n=9*	*Chronic pancreatitis n=22*			
Fibrosis	Fibrosis			
Mild	Absent	Mild	Moderate	Severe
1	0	3	6	8
Inflammation	Inflammation			
Absent	Absent	Mild	Moderate	Severe
0	0	4	9	4
ADM	ADM			
Absent	Absent	Mild	Moderate/severe	
0	4	4	9	

Table 4 summarizes histopathology score of human chronic pancreatitis tissue. Histopathology scores were determined in human chronic pancreatitis tissue for the severity of fibrosis, inflammation and ADM. The control tissues showed not notable histopathology. All 17 CP tissues exhibited fibrosis and inflammation, as well as few ADMs at different severity levels. Severe fibrosis was found in 8 patients, while 6 patients show moderate and 3 mild fibrosis. Histopathology score from control patients showed summarized no tissue injury.
