# Supplementary material for: The bile acid receptor FXR attenuates acinar cell autophagy in chronic pancreatitis
Source: Cell Death Discov. 2017 Jun 19;3:17027–. doi: 10.1038/cddiscovery.2017.27 (PMC5475417; doi:10.1038/cddiscovery.2017.27)
Supplement: Supplementary Figure Legend [file cddiscovery201727-s1.docx]

**Supplemental Figure Legend**

**Supplemental Fig. A. Bile acid GCDC reduces ATG7.** Representative IF FACS-like quantitation of ATG7 in human BxPc-3 cells exposed to 400 µM GCDC with or without (z)-Guggulsterone (GS). ATG7 expression values obtained from were plotted as means ± SEM for three independent experiments as indicated in the graphs. **) *p<*0.01

**Supplemental Fig. B. Bile acid GCDC increases FXR mRNA and reduces ATG7 and ATG5 mRNA.** Human MIA PaCa-2 cells exposed to GCDC induced FXR mRNA and reduces the mRNA levels of autophagic ATG7 and ATG5. Values were determined by real-time PCR and normalized ∆∆CT were plotted as means ± SEM, *) *p*≤0.05.

**Supplemental Fig. C. Bile acid GCDC increases Fxr mRNA and reduces Atg7 and Atg5 mRNA.** AR42J cells exposed to GCDC induced Fxr mRNA and reduces the mRNA levels of autophagic Atg7 and Atg5. Values were determined by real-time PCR and normalized ∆∆CT were plotted as means ± SEM, *) *p*≤0.05.

**Supplemental Fig. D. p62 expression in acinar cells of human pancreatic tissue.** Pancreatic p62 was determined by FACS-like IF quantitation, stained for DAPI (blue) and p62 (green). Representative shown IF images and scattergrams (20x objective; Scale bar = 50 µm). p62 expression values were plotted as means ± SEM for the numbers of patients indicated in the graphs.

**Supplemental Fig. E. Reduced mRNA levels of autophagic factors such as ATG7, ATG5, LC3 and BECLIN-1 in chronic pancreatitis tissue.** Values were determined by real-time PCR and normalized ∆∆CT were plotted as means ± SEM, **) p<0.01.

**Supplemental Fig. F. Cytoplasmic FOXO3 is increased in human chronic panceatitis tissue.** Representative IF FACS-like quantitation of Cytoplasmic FOXO3 in human pancreatic tissue stained for DAPI (blue) and FOXO3 (green). Representative scattergram quantitation from control and chronic pancreatitis patients (20x objective; Scale bar = 50 µm). Cytoplasmic FOXO3 expression values were plotted as means ± SEM for the numbers of patients indicated in the graphs; *) p<0.05.

**Supplemental Fig. G. Human MLKL is increased in human chronic pancreatitis tissue.** MLKL was determined by Immunoblot analysis using the ratio of MLKL and ERK1/2, as indicated by the representative SDS-PAGE autoradiograph. Ratios were plotted as means ± SEM for the numbers of animals indicated in the graphs; *) p<0.05.
