# Supplementary figures and images for: The bile acid receptor FXR attenuates acinar cell autophagy in chronic pancreatitis
Source: Cell Death Discov. 2017 Jun 19;3:17027–. doi: 10.1038/cddiscovery.2017.27 (PMC5475417; doi:10.1038/cddiscovery.2017.27)

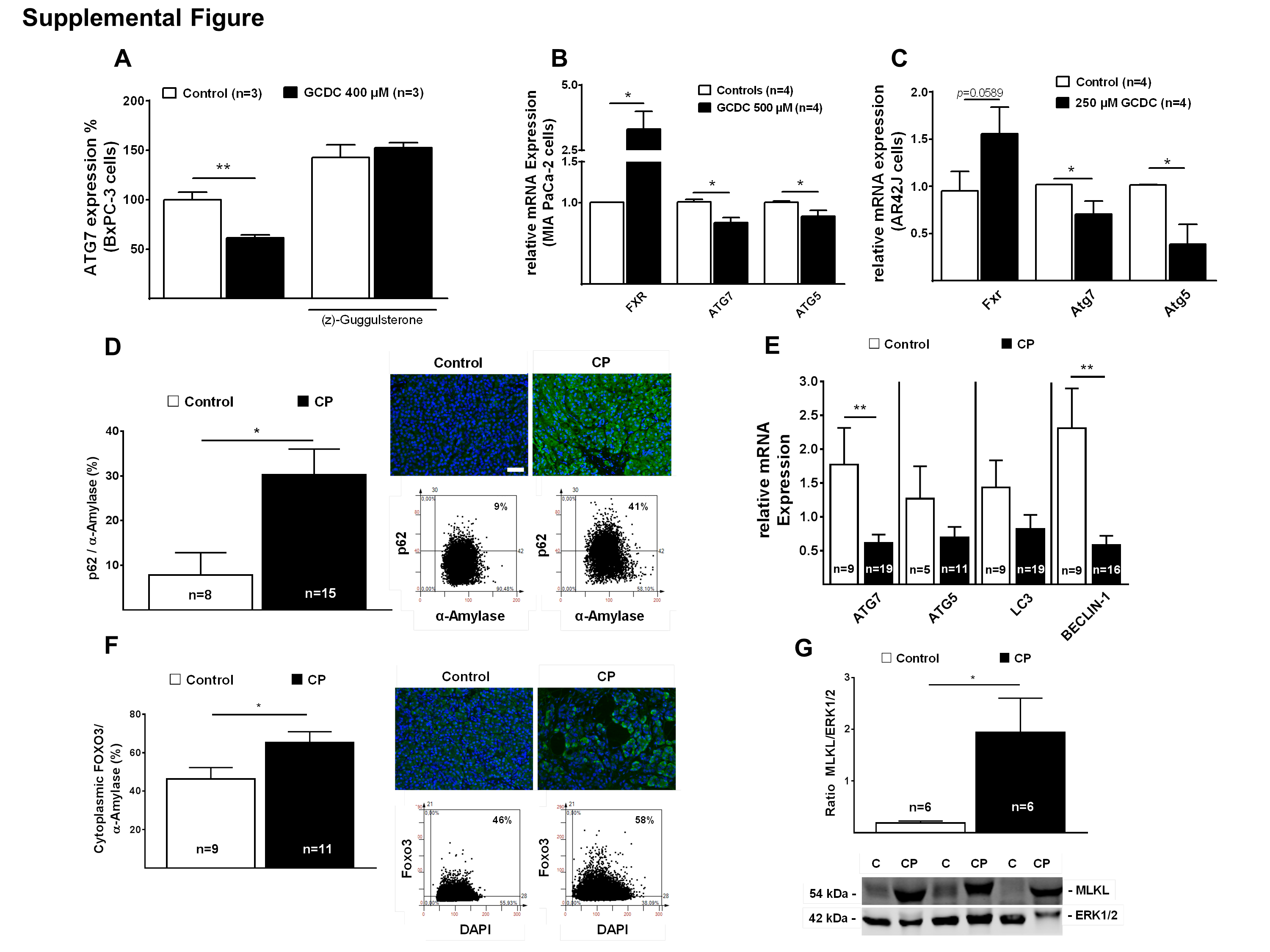

Supplement: Supplementary Figure [file cddiscovery201727-s2.tiff]
